# Impact of Amyloid Burden on Regional Functional Synchronization in the Cognitively Normal Older Adults

**DOI:** 10.1038/s41598-017-15001-8

**Published:** 2017-10-31

**Authors:** Dong Woo Kang, Woo Hee Choi, Won Sang Jung, Yoo Hyun Um, Chang Uk Lee, Hyun Kook Lim

**Affiliations:** 1 Department of Psychiatry, Seoul St. Mary’s Hospital, College of Medicine, The Catholic University of Korea, Seoul, Republic of Korea; 2 Department of Radiology, St. Vincent’s Hospital, College of Medicine, The Catholic University of Korea, Suwon, Republic of Korea; 3 Department of Psychiatry, St. Vincent’s Hospital, College of Medicine, The Catholic University of Korea, Suwon, Republic of Korea; 4 Department of Psychiatry, Yeoui-do St. Mary’s Hospital, College of Medicine, The Catholic University of Korea, Seoul, Republic of Korea

## Abstract

Previous studies have shown aberrant functional connectivity in preclinical Alzheimer’s disease (AD). However, the effects of beta-amyloid (Aβ) retention on regional functional synchronization in cognitively normal older adults still remain unclear. The aim of this study was to explore the distinctive association pattern between Aβ retention and regional functional synchronization in cognitively normal older adults. Sixty-one older adults with normal cognition underwent functional magnetic resonance imaging and regional functional synchronizations were quantified using regional homogeneity (ReHo). Subjects were dichotomized using ^18^F-Florbetaben positron emission tomography imaging into subjects with (Aβ+; n = 30) and without (Aβ-; n = 31) Aβ burden. The Aβ+ group exhibited significantly higher ReHo in the fusiform gyrus and lower ReHo in the precuneus compared with the Aβ- group. We found significant negative correlations between global Aβ retention and ReHo in the precuneus and medial prefrontal cortex and positive correlations between global Aβ retention and ReHo in the bilateral lingual gyrus, left fusiform gyrus, and right middle temporal gyrus in the Aβ+ group. Our findings suggest that regional functional synchronization might have distinctive association patterns with Aβ retention in the cognitively normal older adults. These findings can enrich the functional characterization of early stages of disease progression in AD.

## Introduction

Beta-amyloid (Aβ) deposition plays an important role in the pathogenesis of Alzheimer’s disease (AD). Approximately one third of cognitively normal elderly adults are reported to have some evidence of Aβ deposition^1–4^. In the cognitively normal older adults with Aβ deposition, there is an increased risk of longitudinal cognitive decline and conversion to the symptomatic AD phenotype^5–10^. However, Aβ deposition has been suggested to be necessary, but not sufficient, for the development of AD pathogenesis. Indeed, additional events must occur before cognitive decline and the progression to AD^11^. In this regard, attention has been focused on mediating factors such as functional brain changes occurring in between Aβ deposition and cognitive decline.

Resting state functional MRI (fMRI) reveals spontaneous neuronal activity of the human brain in the resting state and measures useful parameters in evaluating the elderly who have difficulty performing tasks^12,13^. Several prior studies have shown that aberrant functional connectivity (FC) within intrinsic functional networks such as the default mode network (DMN) and central executive network reflected the progression of pathology in the clinical spectrum of AD^14,15^. Although FC abnormalities within the intrinsic functional networks can show integrative pathological changes between two or more discrete brain regions, they cannot identify the particular region responsible for clinical symptoms. To overcome these methodological limitations of FC, regional homogeneity (ReHo) has been developed to evaluate regional resting state brain activity. ReHo is evaluated by Kendall’s coefficient of concordance (KCC) to determine the degree to which the time series of a given voxel is synchronized with its neighbors^16,17^. ReHo is based on the assumption that when brain activity is initiated, it is activated in the form of a cluster rather than a single voxel. Furthermore, ReHo has been reported to reflect intra-regional synchronization, to reveal unpredicted regions and to be more sensitive to the default mode network than other parameters including FC^17,18^. In these regards, several prior studies have evaluated ReHo changes along the AD continuum. In individuals with mild cognitive impairment (MCI), ReHo was decreased in the medial prefrontal cortex, bilateral posterior cingulate cortex and precuneus, but increased in the left inferior parietal lobule, right fusiform gyrus and bilateral putamen compared with cognitively normal elderly^19,20^. In AD patients, ReHo was decreased in the medial prefrontal cortex, bilateral posterior cingulate cortex, and precuneus, but increased in the bilateral cuneus, left lingual gyrus, and right fusiform gyrus^19,21^. These regions are similar to the regions of the default mode network (DMN) and the compensatory region. Moreover, the value of ReHo showed a positive correlation with episodic memory functions^19,21^. In this regard, ReHo has been postulated to act as a noninvasive biomarker reflecting the progression of AD^19^. However, only a few studies have been conducted and were limited by a small number of subjects and lack of identification of Aβ deposition. Although numerous studies have reported the detrimental effect of Aβ deposition on bimodal FC and glucose metabolism in cognitively normal older adults, no study has examined the impact of Aβ deposition on ReHo in cognitively normal elderly^22,23^.

The aim of our study was to determine the impact of Aβ deposition on ReHo in cognitively normal older adults. To distinguish the effects of normal aging from the effects of older subjects at risk of AD, we examined group differences of ReHo between cognitively normal older adults with (Aβ+) and without (Aβ−) Aβ deposition. Moreover, we attempted to explore the association between Aβ deposition and ReHo in the Aβ+ group and evaluated the relationships between ReHo and memory performance in the Aβ+ group versus the Aβ− group. Finally, we explored the sensitivity and specificity of the mean ReHo values in discriminating between the Aβ+ and Aβ− groups.

As previous studies have reported a decreased ReHo index in regions of the DMN during the progression of AD^19^, we hypothesized that ReHo of the Aβ+ group would be substantially decreased when compared with that of Aβ− group. Furthermore, we expected that Aβ deposition would be negatively associated with ReHo in the Aβ+ group. In addition, we hypothesized that there would be significant differences between the Aβ+ and Aβ− groups in the association between episodic memory performance and ReHo maps. Finally, we postulated that mean ReHo values would have significant sensitivity and specificity for discriminating between the Aβ+ and Aβ− groups.

## Results

### Baseline demographic and clinical data

Table 1 shows the baseline demographic data for the two subject groups. All variables were normally distributed. There were no significant differences in gender, age, and education between the Aβ+ and Aβ− groups. In addition, there were no significant differences between the Aβ+ and Aβ− groups on neuropsychological tests performance (Table 1).

**Table 1:** Demographic and clinical characteristics of the study participants.

	*A*β+ group (N = 30)	*A*β− group (N = 31)	P value
Age (years ± SD)	70.2 ± 4.1	69.1 ± 3.7	NS
Education (years ± SD)	9.4 ± 3.2	9.3 ± 4.1	NS
Gender (M:F)	10: 20	12: 19	NS
CDR (SD)	0	0	
**CERAD-K Battery (SD)**			
VF	13.3 ± 3.9	13.2 ± 3.9	NS
BNT	12.7 ± 2.1	12.1 ± 2.1	NS
MMSE	28.4 ± 1.5	28.2 ± 3.1	NS
WLM	18.5 ± 4.5	17.9 ± 3.2	NS
CP	9.4 ± 1.5	9.2 ± 1.9	NS
WLR	7.7 ± 1.8	7.5 ± 1.8	NS
WLRc	9.9 ± 1.2	9.7 ± 1.4	NS
CR	6.7 ± 2.9	6.5 ± 2.3	NS

Aβ+ = cognitively normal older adults with beta amyloid retention, Aβ− = cognitively normal older adults without beta amyloid retention; SD, standard deviation; CDR, Clinical Dementia Rating; CERAD-K, the Korean version of Consortium to Establish a Registry for Alzheimer’s Disease; VF, verbal fluency; BNT, 15-item Boston Naming Test; MMSE, Mini Mental Status Examination; WLM, word list memory; CP, constructional praxis; WLR, word list recall; WLRc, word list recognition; CR, constructional recall.

### Voxel wise FBB PET analysis

**Figure 1:**
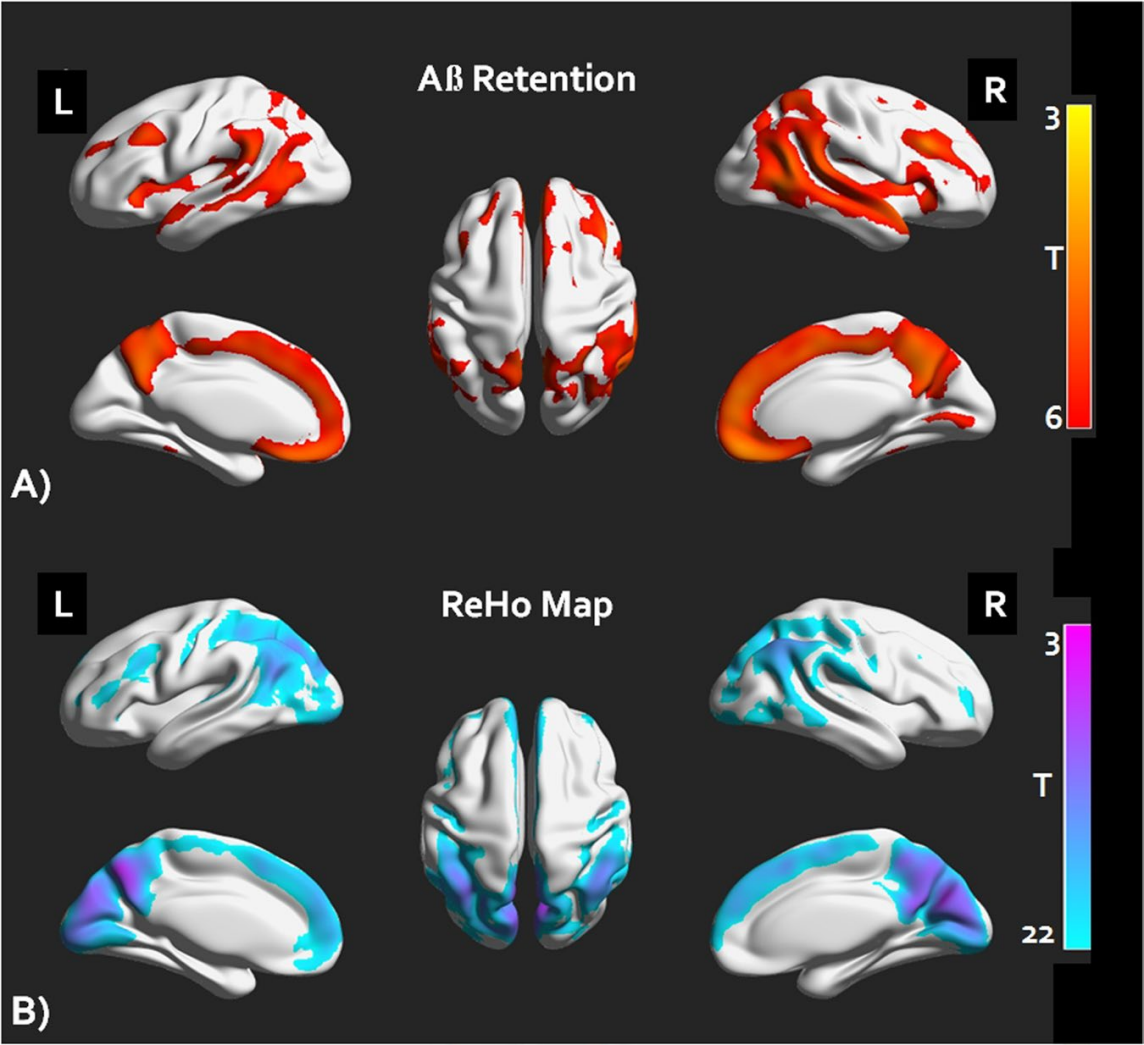
FBB retention pattern and ReHo map of the cognitively normal older adults with amyloid burden (Aβ+) and without amyloid burden (Aβ−). (**A**) A voxel wised group comparison analysis results of amyloid retention between the Aβ+ and the Aβ− group (FDR corrected P < 0.05). (**B**) One sample T test of the ReHo maps of the study participants (Alphasim corrected P < 0.001) FBB = 18F-florbetaben, Aβ = beta amyloid, FDR = false discovery rate, ReHo = regional homogeneity.

The Aβ+ group showed significantly higher retention of Aβ in the anterior cingulate cortex, precuneus, middle frontal cortex and lateral temporal cortex compared to the Aβ− group (Fig. 1A), false discovery rate corrected P < 0.05).

### Within-group and between-group ReHo analyses

**Figure 2:**
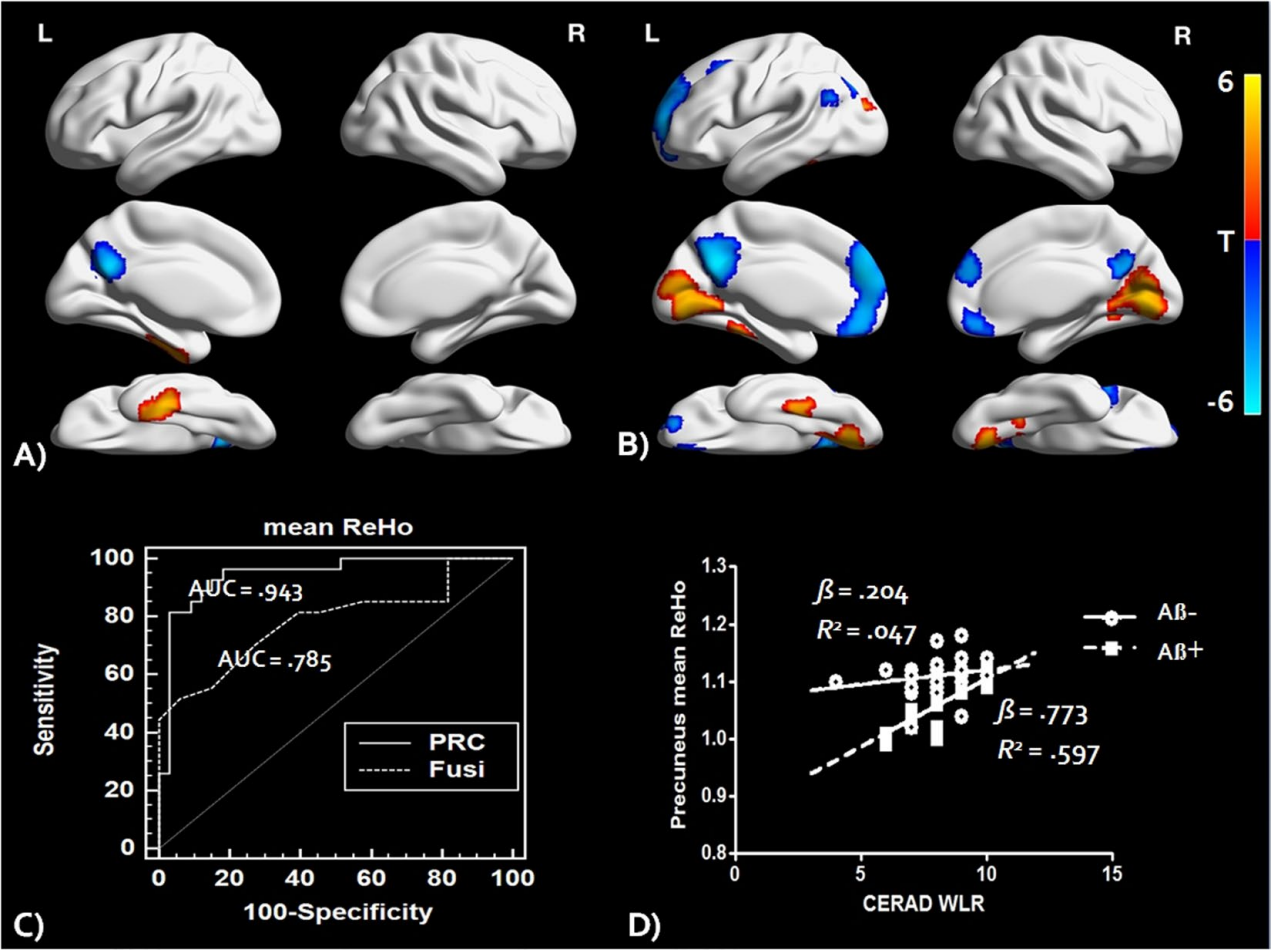
(**A**) A whole brain voxel wise group comparison analysis results of ReHo maps between the Aβ+ and the Aβ− group (Alphasim corrected P < 0.001). (**B**) A whole brain voxel wise correlation analysis results between ReHo values and mean FBB SUVR of the Aβ+ group (Alphasim corrected P < 0.001). (**C**) ROC analysis results of mean ReHo values of left precuneus (PRC) and fusiform (Fusi) in discriminating the Aβ+ from the Aβ− group. (**D**) Mean ReHo values of the left PRC showing a significant interaction between groups in the episodic memory performances (CERAD-WLR scores). ReHo = regional homogeneity, Aβ = beta amyloid, FBB = 18F-florbetaben, ROC = receiver operating curve, CERAD = The Consortium to Establish a Registry for Alzheimer’s Disease, WLR = word list recall, AUC = area under curve.

The mean ReHo maps within each group are shown in Fig. 1B). We found that the posterior cingulate cortex/precuneus, medial prefrontal cortex, and anterior cingulate cortex had high ReHo values within each group (AlphaSim corrected P < 0.001). Compared with the Aβ− group, the Aβ+ group showed a significant ReHo decrease in the left precuneus and increase in the left fusiform gyrus (Table 2, Fig. 2A)), AlphaSim corrected P < 0.001). The mean ReHo values from these regions of interest (ROIs) were used for ROC (receiver operator characteristic analysis) in discriminating the Aβ+ group from the Aβ− group (Fig. 2C)). The mean ReHo value of the left precuneus showed an AUC (area under curve) value of 0.94, sensitivity of 82% and specificity of 97%, whereas the mean value of the left fusiform gyrus showed an AUC value of 0.79, sensitivity of 51% and specificity of 93% in discriminating the Aβ+ group from the Aβ− group. In addition, we also investigated the effect of Aβ on the relationships between episodic memory and the mean ReHo values of these ROIs. There was a significant group by episodic memory (The Consortium to Establish a Registry for Alzheimer’s Disease (CERAD) Word List Recall (WLR) scores) interaction for the mean ReHo value of the left precuneus (F = 10.29, P < 0.0001, Fig. 2D), however there was no significant interaction for the mean ReHo value of the fusiform gyrus.

**Table 2:** Whole brain Voxel wise ReHo analysis results.

Region	L/R	Cluster	P value*	MNI (x,y.z)			
**Group Differences**							
*Aβ*+> *A*β−							
Fusiform gyrus	L	180	<0.001	−36		−18	−30
*Aβ*+***< *** *A*β−							
Precuneus	L	118	<0.001	−9		−57	27
**Mean FBB SUVR-ReHo correlations in** ***Aβ*** **+ group**							
*Negative correlation*							
Superior medial frontal gyrus	L	401	<0.001	−9		51	21
Superior medial frontal gyrus	R	132	<0.001	10		53	27
Medial orbitofrontal gyrus	R	121	<0.001	2		45	−22
Precuneus	L	189	<0.001	−9		−57	30
Precuneus	R	107	<0.001	5		−59	26
Angular gyrus	L	89	<0.001	−40		−63	27
*Positive correlation*							
Lingual gyrus	R	296	<0.001	9		−75	3
Lingual gyrus	L	271	<0.001	−8		−75	−1
Fusiform gyrus	L	112	<0.001	−36		−37	−28
Middle temporal gyrus	R	82	<0.001	46		−25	−10

*Alphasim corrected P < 0.001 values for the multiple comparisons. ReHo = regional homogeneity, Aβ+ = cognitively normal older adults with beta amyloid retention, Aβ− = cognitively normal older adults without beta amyloid retention, FBB = 18F-Florbetaben, SUVR = standardized uptake value ratio, MNI = Montreal Neurological Institute coordinate.

### Correlations between ReHo and Aβ deposition

Figure 2(B) and Table 2 show correlation analysis results between the global mean Florbetaben (^18^F)(FBB) retention and ReHo values in the Aβ+ group alone. In the Aβ+ group, ReHo in the left and right superior medial frontal gyrus, precuneus, left orbitofrontal cortex and left angular gyrus showed a significant negative correlation with the global FBB retention (AlphaSim corrected P < 0.001). In addition, ReHo in the left and right lingual gyrus, left fusiform gyrus and right middle temporal gyrus was positively correlated with the global FBB retention in the Aβ+ group (AlphaSim corrected P < 0.001).

### Mediation Analysis

**Figure 3:**
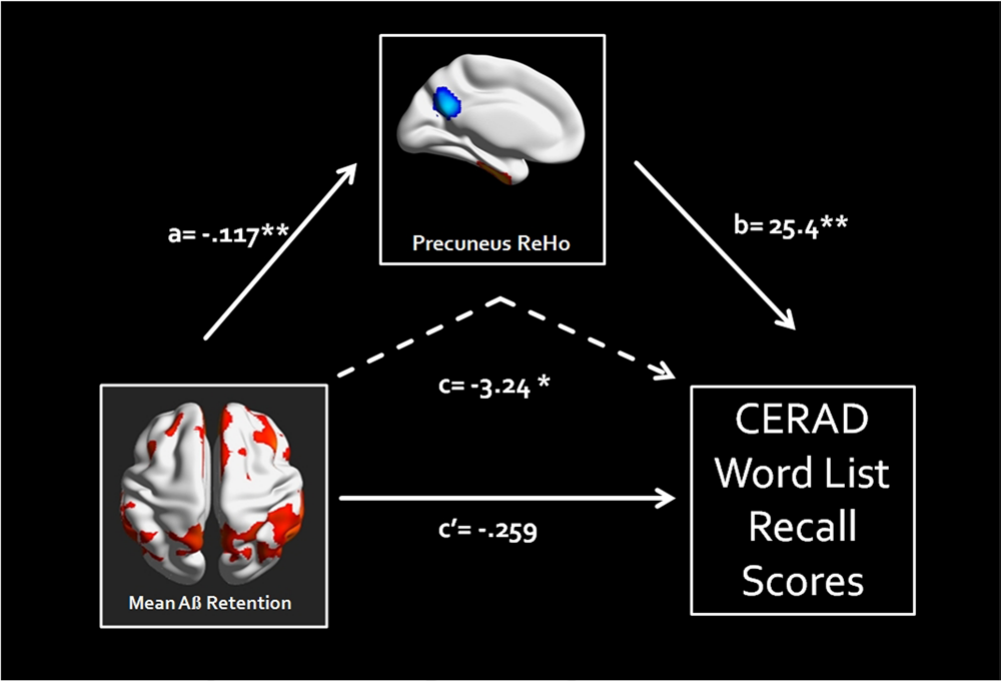
Mediation Model of the left precuneus mean ReHo values between Aβ retention and episodic memory performance (CERAD WLR scores) in the Aβ+ group. As indicated by the path coefficient and p-value, path c is the total effect of Aβ retention on the episodic memory performance, path c’ is the direct effect of the Aß retention on the episodic memory performance *p < 0.001, **p < 0.0001, ReHo = regional homogeneity, Aβ = beta amyloid, CERAD = The Consortium to Establish a Registry for Alzheimer’s Disease, WLR = word list recall.

Figure 3 shows the results of mediation analysis with the global mean FBB SUVR values as an independent factor and CERAD WLR scores as dependent factors in the Aβ+ group. The proposed mediator was the mean ReHo value of the left precuneus which showed significant group by episodic memory function as indicated above. The mediation analysis showed that there was no significant direct effect of global mean FBB SUVR values on CERAD WLR scores (β = −0.15, p = 0.37). On the other hand, the effect of global mean FBB SUVR values on CERAD WLR scores was mediated by the left precuneus mean ReHo values (β = −0.57, p = 0.008).

## Discussion

To the best of our knowledge, this is the first study to determine the impact of Aβ burden on regional functional synchronization in cognitively normal older adults. A key strength of the present study was the measurement of Aβ retention by amyloid positron emission tomography (PET) for differentiating cognitively normal older adults at risk of AD from normal controls. Hence, we could conduct a more thorough investigation on the relationships between the degree of Aβ retention and the ReHo value as well as differences in ReHo between cognitively normal subjects at risk of AD and healthy controls to clarify changes in intra-regional brain activity during the earliest phase of AD.

The current study found that regions with higher Aβ retention in the Aβ+ group overlapped with regions with higher ReHo in both groups. These overlapping regions were consistent with the components of the DMN including the anterior cingulate cortex and precuneus^24^, which is in agreement with a previous study which showed a similar distribution of higher ReHo in cognitively normal older adults and AD subjects^21^. To date, several prior works showed that topographical distributions of Aβ retention appeared to overlap the regions within the DMN, which has a higher basal metabolic activity, compared with the other brain regions^22,25^. Furthermore, the positive relationship between Aβ retention and intrinsic FCs has been shown at the global network level from the earliest stage of AD^26^. In this regard, Aβ retention has been suggested to be accelerated^25,27,28^ and preceded^29,30^ by increased metabolism, intrinsic activity and connectivity. Collectively, we could infer that the pattern of ReHo reflecting intra-regional brain activity would be similar to that of resting metabolic activity in the DMN, with overlapped regions of Aβ retention at the earliest stage of AD.

We also found that the Aβ+ group showed lower ReHo in the left precuneus compared with the Aβ− group. This result is in line with previous studies that showed lower ReHo in the posterior cingulate cortex/precuneus in MCI and AD compared with control group^19–21^. However, the findings of our study do not support earlier investigations using fluorodeoxyglucose (FDG)-PET, which have demonstrated non-significant differences of regional metabolism between cognitively normal subjects with and without Aβ retention^31^. Furthermore, the current study found a significant negative correlation between the global Aβ retention and the ReHo within the DMN regions including precuneus in the Aβ+ group. This is also in accordance with earlier observations, which showed that the negative impact of Aβ retention on FCs and local metabolic activity in the posterior DMN of cognitively normal adults with Aβ deposition^26,32–34^. However, this finding appears to be contrary to aforementioned results which have suggested that heightened intrinsic brain activity may induce amyloid retention. A possible explanation for this discrepancy may be that Aβ retention has the driving force of reducing the metabolic activity only after extensive Aβ deposition induced by higher basal metabolism^35^. In addition, the reduction of intra-regional metabolic activity has been demonstrated to initiate from the earliest phase of AD and to peak at the prodromal stage of AD^26^. Therefore, an intra-regional brain activity measured by the cross-sectional analyses in cognitively normal subjects at risk of AD could be affected from the extent to which intra-regional brain activity is reduced. Therefore, inconsistent differences of intra-regional brain activity between cognitively normal subjects with and without Aβ retention might be interpreted with the aforementioned proposition.

We found that the Aβ+ group showed higher ReHo in the left fusiform gyrus compared with the Aβ- group. Furthermore, the present study found a significant positive correlation between Aβ retention and ReHo in the bilateral lingual gyrus, left fusiform gyrus and middle temporal gyrus in the Aβ+ group. These results are concordant with the findings of previous studies which showed higher ReHo in the fusiform gyrus in MCI and AD subjects compared to healthy controls^20,21^. Moreover, the higher intra-regional activity of the fusiform gyrus was suggested to be compensatory for the progression of AD pathogenesis^20,21^. This is also in accordance with earlier observations, which showed the positive correlation between the ReHo of compensatory regions (including the fusiform gyrus) and individual intelligence^36^. This finding is also exemplified in the previous research, which have shown that increased Aβ retention is associated with increased functional connectivity of lateral temporal cortices and is suggested to be compensatory^32^.

Another important finding was that the mean ReHo values of the left precuneus showed higher AUC, sensitivity and specificity in discriminating the Aβ+ group from the Aβ− group. In previous research, the mean ReHo values could discriminate subjects based on the stage of cognitive impairment (normal control, MCI, or AD) at a rate that was 71.4% correct^19^. Taken together, these results strengthen the idea that the mean ReHo value might be sensitive to earlier changes in spontaneous brain activity in response to Aβ retention. However, further longitudinal study with larger sample size would be needed to confirm the ReHo as a potential functional biomarker for predicting the risk of AD.

In the current study, we also found significant group × memory retrieval interactions in the left precuneus. Furthermore, both groups showed a positive correlation between the ReHo and memory retrieval in the left precuneus. These findings are consistent with that of previous study, which have shown the positive relationship between ReHo of the posterior cingulate cortex/precuneus and scores of mini mental status examination in the AD patient group^21^. The precuneus/posterior cingulate cortex has been documented to activate during memory retrieval^37^, to network with the medial temporal lobe and to engage in memory function in the early phase of AD^25^. In addition, these results are in line with our previous study showing group × episodic memory interactions in the posterior DMN FC and positive correlations between posterior DMN FC and episodic memory function scores in the cognitively normal older adults with Aβ retention^34^. Therefore, our results with positive correlation between the precuneus ReHo and memory retrieval suggest the downstream effect of the Aβ deposition on the left precuneus ReHo, where lower functional synchronization is associated with poorer episodic memory^38^. However, these simple association patterns are not sufficient to clarify the role of the ReHo linking Aβ retention and cognitive impairments, which are essential characteristics of AD^4^. In these regards, we conducted a mediation analysis to clarify whether the left precuneus ReHo mediated the link between Aβ deposition and episodic memory impairments. Interestingly, the mediation analysis results showed that the left precuneus ReHo completely mediated the link between Aβ retention and cognitive impairments. These results may help to explain the inconsistent results of association between Aβ retention and cognitive impairments in the cognitively normal older adults^31,32,34^. In addition, our results also expands several prior works showing mediation effects of the DMN FCs and brain glucose metabolism between Aβ retention and cognitive impairments in the cognitively normal^39,40^ and the prodromal AD subjects^41^. However, further longitudinal prospective studies will be necessary to confirm mediation effects of the ReHo between Aβ retention and cognitive impairments on the trajectories of AD.

The major limitation of this study is the use of a cross-sectional design. The cross-sectional design precludes our ability to make causal inferences; however, it allows us to generate hypotheses for future studies. Therefore, these cross-sectional findings require validation in prospective studies. Another limitation includes the lack of examination of the apolipoprotein epsilon4 (APOE4) allele and tau pathology which has been reported to be associated with brain activity in the DMN in cognitively normal younger and older adults^42–44^. Therefore, further longitudinal, controlled research should be conducted in order to determine the causal effect of Aβ retention on ReHo, or vice versa.

In summary, this study identified alterations in ReHo, differential patterns of association between Aβ deposition and ReHo, the relationship between memory retrieval and ReHo in cognitively normal older adults with Aβ retention. Moreover, the features of ReHo in discriminating cognitively normal older subjects at risk of AD from those in normal aging process. This combination of findings provides some support for the value of intra-regional brain activity for understanding the earliest pathogenesis of AD. However, more longitudinal research on this topic needs to be undertaken in conjunction with other parameters for clarifying the role of ReHo in the progression of AD.

## Methods

### Subjects

Sixty one elderly subjects with normal cognition were included in this study. They were recruited from the normal control volunteers of the Catholic Dementia Brain Imaging Database, which holds brain scans of outpatients and inpatients at the Department of Geriatric Psychiatry, Saint Vincent Hospital, The Catholic University of Korea from 2010 to 2016. The inclusion criteria of the subjects were as follows: (i) subjects aged ≥60 years; (ii) Mini-Mental Status Examination score ≥27; and (iii) Clinical Dementia Rating = 0^45^. Subjects with any psychiatric, neurological and unstable medical conditions were excluded. The cognitive testing battery included the following domains: memory, visuospatial construction, language, attention and executive functions. Details on the specific tests used and the reviewing process are described in the Supplementary material. The study was conducted in accordance with the ethical and safety guidelines set forth by the local Institutional Review Board of the Catholic University of Korea and written informed consent was obtained from all study subjects. The local Institutional Review Board of the Catholic University of Korea approved this study (No. VC15EISI0044) following the principles set forth by the Declaration of Helsinki.

### PET acquisition

FBB was produced and FBB-PET data were collected and analyzed as previously described^46^. Each individual participant’s MRI was utilized for co-registration and defining the ROI and for correcting partial volume effects from expanding cerebrospinal spaces accompanying cerebral atrophy^47,48^. Analysis of the FBB PET data utilized a standardized uptake value ratio (SUVR) 90 min post-injection, using the cerebellar cortex region of interest as the reference. Global Aβ burden was expressed as the average SUVR of the mean for the following 5 cortical ROIs: frontal, superior parietal, lateral temporal, and anterior and posterior cingulate cortex/precuneus as described in previous study^48^.

The FBB PET data were acquired within 4 weeks of clinical screening and cognitive function test. We used a cut-off for ‘high’ or ‘low’ neocortical SUVR of 1.4, consistent with cut-off values used in previous FBB-PET study^49^. PET image preprocessing and voxel wise FBB PET analysis processes are described in detail in the Supplementary material.

### MRI acquisition

Imaging data were collected at the Department of Radiology, St Vincent’s Hospital, The Catholic University of Korea, using a 3T Siemens Verio machine and eight channel Siemens head coil(Siemens Medical Solutions, Erlangen, Germany). The parameters used for the T1-weighted volumetric magnetization-prepared rapid gradient echo scan sequences were TE = 2.5 ms, TR = 1,900 ms, inversion time = 900 ms, FOV = 250 mm, matrix = 256 × 256, and voxel size = 1.0 × 1.0 × 1.0 mm^3^. Resting state functional images were collected using a T2* weighting gradient echo sequence with TR = 2,490 ms, TE = 30 ms, matrix = 128 × 128 × 29, and voxel size = 2 × 2 × 3 mm^3^. One hundred and fifty volumes were acquired over 5 minutes with the instruction “keep your eyes closed and think of nothing in particular”.

### Data analysis

We used the Data Processing Assistant for Resting-State fMRI (DPARSF)^50^ which is based on Statistical Parametric Mapping (SPM, http://www.fil.ion.ucl.ac.uk/spm) to preprocess the fMRI images. Slice timing and realignment for motion corrections were performed on the images. Subjects with excessive head motion (cumulative translation or rotation > 2mm or 2°) were excluded. To prevent group-related differences from micro-head motion, framewise displacement (FD) was compared between the groups. Mean FD scores were not different between the groups (P > 0.05, two- sample t-tests) and further used as covariates in group comparisons. In spatial normalization, the International Consortium for Brain Mapping (ICBM) template was applied (resampling voxel size = 3mm × 3mm × 3mm) which was fitted to the ‘East Asian brain’.

We further processed our functional data to make them fit for ReHo analysis through the Data Processing Assistant for Resting-State fMRI^50^. Above all, linear trends were removed from the functional images. After this, the data were filtered with a temporal band-pass of 0.01–0.08 Hz. This filtering reduces low- frequency drift as well as physiological high-frequency respiratory and cardiac noise^12^. Next, ReHo maps of all participants were made by a general routine using DPARSF. Briefly, we set the basic cube to calculate the KCC by 3mm × 3mm × 3mm voxels. Therefore, the KCC value of the central voxel in the cube was calculated by referring to the temporal sequences of its neighboring 26 voxels. The calculated value was assigned as the ReHo value of the central voxel. This calculation was repeated for all the voxels throughout the brain; subsequently, an unsmoothed ReHo map was drawn. This raw ReHo map was smoothed by 6mm of full width at half maximum (FWHM). This smoothing is appropriate for the cluster-level analysis which is described in the next section^51,52^.

### Statistical analysis

Statistical analyses for demographic data were performed with the Statistical Package for Social Sciences software (SPSS, version 12.0, Chicago, IL). The independent t-test and the χ^2^ test were used to assess potential differences between the Aβ+ and the Aβ− groups for all demographic and clinical variables and SUVR values. All statistical analyses used a two-tailed level of 0.05 for defining statistical significance. The general linear model (GLM) was used for measuring the within and between group differences of the ReHo maps. To examine the relationships between Aβ deposition and ReHo in the Aβ+ group, the global mean SUVR value from the 5 ROIs were correlated with the voxel-wise ReHo maps of the brain using GLM.

Mean ReHo values from brain regions with significant group differences were used further ROIs analysis. We performed binary logistic regression with ROC analysis to evaluate the sensitivity and specificity of the mean ReHo values from the ROIs to discriminate the Aβ+ group from the Aβ− group. In addition, the GLM with ReHo as the dependent variable Word List Recall (CERAD WLR) scores and group as the independent variables were performed, as well as analysis on the interaction (episodic memory × group). We controlled for the effect of age, education and gender in all GLM analysis used.

Multiple corrections were performed using cluster-extent correction (AlphaSim) as implemented through DPABI, and the parameters were set as follows: individual voxel threshold p < 0.001, number of Monte Carlo simulations = 1000, and p = 0.001 as the effective threshold for cluster-extent correction.

Furthermore, to address the question of whether the regional functional synchronization mediated the association between Aβ retention and episodic memory performances in the Aβ+ group, a mediation analysis was performed using the PROCESS macro^53^ controlled for age, sex, and education level.

### Data Availability

The datasets generated and analyzed during the current study are available from the corresponding author on reasonable request.

## Electronic supplementary material

supplemental materials
