# Supplementary material for: Impact of Amyloid Burden on Regional Functional Synchronization in the Cognitively Normal Older Adults
Source: Sci Rep. 2017 Oct 31;7:14690. doi: 10.1038/s41598-017-15001-8 (PMC5665874; doi:10.1038/s41598-017-15001-8)
Supplement: Supplementary file 1 — supplemental materials [file 41598_2017_15001_MOESM1_ESM.doc]

**Impact of Amyloid Burden on Regional Functional Synchronization in the Cognitively Normal Older Adults**

Dong Woo Kang ^1^, Woo Hee Choi^2^, Won Sang Jung^2^, Yoo Hyun Um ^3^, Chang Uk Lee^1^, Hyun Kook Lim^4^*

^1^ Department of Psychiatry, Seoul St. Mary’s Hospital, College of Medicine, The Catholic University of Korea, Seoul, Republic of Korea

^2^Department of Radiology, St. Vincent’s Hospital, College of Medicine, The Catholic University of Korea, Suwon, Republic of Korea

^3^Department of Psychiatry, St. Vincent’s Hospital, College of Medicine, The Catholic University of Korea, Suwon, Republic of Korea

^4^Department of Psychiatry, Yeoui-do St. Mary’s Hospital, College of Medicine, The Catholic University of Korea, Seoul, Republic of Korea

*Send all correspondence to

Hyun Kook Lim M.D., Ph.D.

Department of Psychiatry, Yeoui-do St. Mary’s Hospital, College of Medicine, The Catholic University of Korea, 63 Ro 10 Seoul, Republic of Korea

E-mail: drblues@catholic.ac.kr

Phone: +82-2-3779-1048

Fax: +82-2-780-6577

**Supplemental Materials**

***1. Neuropsychological evaluation***

Cognitive status was assessed by neuropsychological testing at the St. Vincent Hospital The Catholic University of KOREA. The cognitive functions of all the subjects were assessed with the Korean version of Consortium to Establish a Registry for Alzheimer’s Disease (CERAD-K), including Verbal Fluency (VF), 15-item Boston Naming Test (BNT), MMSE, Word List Memory (WLM), Word List Recall (WLR), Word List Recognition (WLRc), Construc- tional Praxis (CP), and Constructional Recall (CR).

The results were reviewed by a neuropsychologist to determine whether there was evidence of cognitive impairment. The criteria was not dictated by strict cutoff scores, but were also clinically guided by the following principles: (a) there was evidence on at least two different tests of performance significantly below expectations given the individual′s age and educational background (scores were generally considered to be in the impaired range if they fell more than 1standard deviation (SD) below age means, taking into account the individual′s level of education); (b) low test scores were supported by reports from the participant with changes or concerns about memory or cognition or by behavioral observations by study staff.

***2. Voxel wise FBB PET analysis***

High-resolution, skull-cropped, 3T MP-RAGE images were reoriented along the AC-PC line and the medial longitudinal fissure. These images were normalized to the ICBM 152 template (Montreal Neurological Institute) using tissue priors and the unified segmentation technique[^1^](#_ENREF_1) of the SPM12 software package (http://www.fil.ion.ucl.ac.uk/spm/software/spm8/). ¹⁸F-Florbetaben (FBB) standard uptake value ratio (SUVR) parametric images were prepared by applying a positive mask, co-registering to the corresponding MR image, and transforming into template space using the factors created during segmentation. A general linear model (GLM) analysis was used for the group comparison between the Aß + and the Aß - groups with age, gender and education controlled. The threshold was set at p<0.05 (false discovery rate (FDR)) to resolve the problem of multiple comparisons.

***3. Region of Interest (ROI) Group Analysis Results***

For the better understanding of the distribution of the value of the ReHo in the left precuneus and fusiform gyrus, we conducted the descriptive statistics and the Kolmogorov-Smirnov test for the normal distribution. In addition, analysis of covariance test (ANCOVA) was conducted for group comparison controlling for age, sex and education level. As shown in Table S1, the data of the ReHo values in the left precuneus and fusiform gyrus were normally distributed in each group. In addition, the ANCOVA analysis revealed that the left precuneus ReHo values were significantly lower in the Aß+ group compared with Aß- group (F=37.1, p<0.0001). On the other hand, the left fusiform gyrus ReHo values were significantly higher in the Aß+ group compared with Aß- group (F=17.4, p<0.0001).

***4. Correlation analysis between the combined ROIs and the Aß retention***.

To understand the impact of the Aß retention on the ReHo values of the combined ROIs, we created the two combined ROIs from the statistical maps of the voxel wise correlation analysis results (Figure 2 B)) between the ReHo and the Aß retention in the Aß+ group, using the MarsBaR toolbox (http://marsbar.sourceforge.net/). The combined negative correlation ROI (CN-ROI) included the left and right superior medial frontal gyrus, the right medial orbitofrontal gyrus, the left and right precuneus, and the left angular gyrus, where voxel wise analysis showed negative correlations between the ReHo and the Aß retention in the Aß+ group. In addition, the combined positive correlation ROI (CP-ROI) included the left and right lingual gyrus, the left fusifom gyrus, and the right middle temporal gyrus, where voxel wise analysis showed positive correlations between the ReHo values and the Aß retention in the Aß+ group. We extracted the ReHo values from these two combined ROIs, and conducted correlation analysis between the ReHo values and the global mean FBB SUVRs, after controlling for age, sex and education. The correlation analysis results showed that the ReHo values of the CN-ROI were significantly negatively correlated with the global mean FBB SUVRs (*r*= -0.45; p=0.001). In addition, the ReHo values of the CP-ROI showed significant positive correlations with the global mean FBB SUVRs in the Aß+ group (*r*= 0.43; p=0.001).

**Table S1. Descriptive statistics of the left precuneus and fusiform gyrus ReHo values used in the region of interest analysis**

|  | ***Aß+* group (N=30)** | ***Aß-* group (N=31)** |
| --- | --- | --- |
| **Left Precuneus** |  |  |
| Mean ReHo | 1.05 | 1.11 |
| Standard deviation | 0.03 | 0.02 |
| 95% Confidence Interval | 1.04-1.06 | 1.10-1.12 |
| Kolmogorov-Smirnov test (p value) | 0.18 | 0.1 |
| **Left Fusiform gyrus** |  |  |
| Mean ReHo | 0.95 | 0.90 |
| Standard deviation | 0.05 | 0.04 |
| 95% Confidence Interval | 0.93-0.96 | 0.89-0.90 |
| Kolmogorov-Smirnov test (p value) | 0.2 | 0.1 |

**References**

1 Ashburner, J. & Friston, K. J. Unified segmentation. *Neuroimage* **26**, 839-851, doi:10.1016/j.neuroimage.2005.02.018 (2005).
